# Evaluating the consistency in different methods for measuring left atrium diameters

**DOI:** 10.1186/s12880-024-01231-6

**Published:** 2024-03-05

**Authors:** Jun-Yan Yue, Kai Ji, Hai-Peng Liu, Qing-Wu Wu, Chang-Hua Liang, Jian-Bo Gao

**Affiliations:** 1 Department of Radiology, The First Affiliated Hospital of Xinxiang Medical University, Weihui Henan Province, 453200 Xinxiang, China; 2 Department of Radiology, The First Affiliated Hospital of Zhengzhou University, No. 1 Jianshe East Road, Erqi District, 450000 Zhengzhou, Henan Province China; 3 Heart Center, The First Affiliated Hospital of Xinxiang Medical University, 453200 Henan Pro vince, Weihui China

**Keywords:** Diameter, Left atrium, Measurement, Tomographic technique, X-ray computer

## Abstract

**Background:**

The morphological information of the pulmonary vein (PV) and left atrium (LA) is of immense clinical importance for effective atrial fibrillation ablation. The aim of this study is to examine the consistency in different LA diameter measurement techniques.

**Methods:**

Retrospective imaging data from 87 patients diagnosed with PV computed tomography angiography were included. The patients consisted of 50 males and 37 females, with an average age of (60.74 ± 8.70) years. Two physicians independently measured the anteroposterior diameter, long diameter, and transverse diameter of the LA using six different methods. Additionally, we recorded the post-processing time of the images. Physician 1 conducted measurements twice with a one-month interval between the measurements to assess intra-rater reliability. Using the intraclass correlation coefficient (ICC), the consistency of each LA diameter measurement by the two physicians was evaluated. We compared the differences in the LA diameter and the time consumed for measurements using different methods. This was done by employing the rank sum test of a randomized block design (Friedman M test) and the *q* test for pairwise comparisons among multiple relevant samples.

**Results:**

(1) The consistency of the measured LA diameter by the two physicians was strong or very strong. (2) There were statistical differences in the anteroposterior diameter, long diameter, and transverse diameter of LA assessed using different methods (*χ*^*2*^ = 222.28, 32.74, 293.83, *P* < 0.001). (3) Different methods for measuring the diameters of LA required different amounts of time (*χ*^*2*^ = 333.10, *P* < 0.001).

**Conclusion:**

The results of left atrium (LA) diameter measurements conducted by different physicians were found to be reliable. However, the LA diameters obtained through various techniques exhibited variations. It was observed that measuring LA long diameters using only the VR (volume rendering) picture was the most clinically applicable method.

## Background

Atrial fibrillation (AF) is a progressive disease with different atrial remodeling symptoms in different stages. In the early stage, it manifests as electrical remodeling, and in the late stage, it manifests as structural remodeling such as atrial fibrosis [1]. 

Numerous echocardiographic studies have demonstrated the predictive value of atrial diameters in the occurrence, progression or recurrence of AF [2–7]. Longitudinal remodeling (increase in vertical diameter), transverse diameter, anteroposterior diameter, and volume of left atrium (LA) are associated with the recurrence of AF after radiofrequency ablation or the occurrence of AF [2–5]. Enlargement of both atriums is an independent predictor of the first ablation following the initial ablation in patients with AF [7]. LA diameters may contribute to identifying patients at high risk for AF [8]. 

Computed tomography (CT) and magnetic resonance imaging (MRI) are commonly utilized in the advanced stages of cardiac disease. The CT examination is simple and easy, but it is not easy to observe the situation of atrial fibrosis, while MRI Examination takes a longer examination time. [9–10] Several studies have independently contributed to the understanding of the relationship between LA diameters and AF [11–13]. The left interior pulmonary vein (PV) direction and LA diameter, LA anteroposterior diameter enlargement, LA volume index, and mean diameter of PV orifice are all associated with the occurrence and recurrence of AF [11–13]. However, it has been observed that the LA volume, LA size, and PV size cannot independently predict the postoperative recurrence of non-paroxysmal AF [14]. According to some studies, there is no morphological or functional sign in heart CT to predict the early recurrence of AF following atrial radiofrequency ablation [15]. 

In our study, all echocardiographic findings were positive. However, CT and MRI results were somewhat positive and negative, and not all CT results were the same although some were comparable, and different methods were used to measure LA diameter [12, 14]. Only the LA volume, but not their diameters was measured in some studies [13]. However, there are other studies where the LA diameters were measured, but the measurement methods or specific images were not provided [11, 15]. Thus, we hypothesize that the different results are attributable to the respective measuring methods.

Therefore, the purpose of this study is to compare and assess different methods for measuring LA diameters in order to identify clinically applicable methods for measuring LA diameters.

## Data and methods

### Participants

The Ethics Committee of the First Affiliated Hospital of Xinxiang Medical University approved this study. From January to December 2021, image data from PV computed tomography angiography (CTA) conducted at the First Affiliated Hospital of Xinxiang Medical University were collected and included in the study.

### Inclusion and exclusion criteria

Inclusion criteria: (1) all patients with PV CTA; (2) patients with complete and qualified imaging data.

Exclusion criteria: (1) patients with low-quality images who are ineligible for CTA remodeling; (2) patients with non-whole-phase scanning who are ineligible for multiphase remodeling.

### CT examination method

The CT machines used were the Canon Aquilion ONE 320 slice CT and GE Revolution 256 slice CT, with the following scanning parameters: 120 kV, auto-milliampere, volume scanning with 1.00 and 1.25 mm slice thickness, and 1.00 and 1.25 mm slice gaps. The required scanning distance was 16 cm, centered on the heart. About 40 ∼ 70 ml of Iopromide Injection 370 (specification: 100 ml: 76.89 g) was administered using a high pressure injector at the rate of 5 ml/s, and the total dose was calculated based on the weight of the patient and drug instructions using the formula: total dose = weight (kg) × (1.0 ∼ 1.5 ml/kg). In general, the CT value of the ascending aorta at the level of the pulmonary artery was monitored 10 s after administration; The CT scanning procedure was initiated when the CT value reached 150 Hounsfield Units (HU), and it could be initiated either automatically or manually. ECG-gating was employed, and the patient was instructed to hold their breath during a normal respiration state prior to the total cardiac cycle scanning. Each patient underwent multi-phase remodeling ranging from 0 to 99% at intervals of 10%, resulting in a total of 10 phases.

### Image processing methods

All multiphase images were forwarded to the Canon Vitrea workstation for post-processing. Images of the end-systole (LA diastole) of the left ventricle were chosen.

**Fig. 1:**
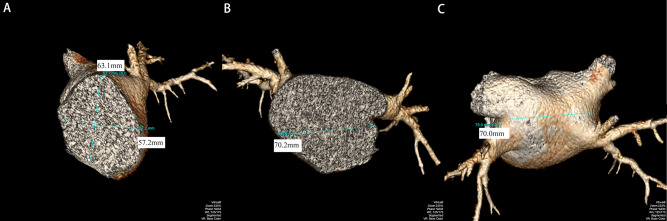
**A**: Measurement of the long and anteroposterior diameters of LA in the median sagittal plane. **B**: Measurement of the transverse diameter of LA at a median coronal plane of LA. **C**: The minimum distance of the extension line between the left and right PV orifices was measured on the VR image to represent the transverse diameter

Volume rendering (VR)① image processing method: VR images of LA were modified independently. The distance from the midpoint of the mitral annulus plane to the dome at the top of LA was measured on a median sagittal plane as the LA long diameter (Fig. 1A); the distance between the anterior and posterior walls of the LA through the midpoint of the long diameter was the LA anteroposterior diameter (Fig. 1A); and the line from the midpoint of the atrial septum to the midpoint of the left lateral wall of LA was measured on a median coronal plane of LA as the LA transverse diameter a (Fig. 1B). The minimum distance of the extension line between the left and right PV orifices was measured on the VR image to represent the transverse diameter (Fig. 1C).

**Fig. 2:**
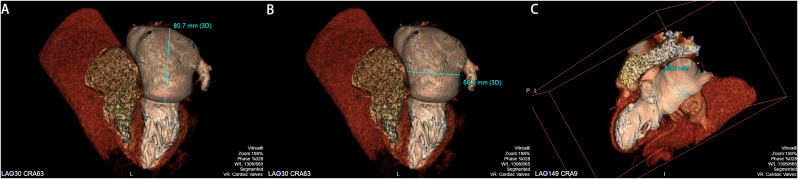
**A**: Measurement of LA long diameter at the apical four-chamber plane. **B**: Measurement of LA transverse diameter at the apical four-chamber plane. **C**: LA anteroposterior diameter measurement along the long axis of the apical two-chamber plane

VR② image processing method: In the Cardiac Valves mode pattern, when the VR image shows LAO was displayed at 30° and CRA was displayed at about 60°, selecting the forward-cut mode, the long diameter (Fig. 2A) and transverse diameter of LA were measured at the apical four-chamber plane (Fig. 2B), and when the VR image shows LAO at 145° and CRA at about 0°, selecting the oblique-cut mode, the anteroposterior diameter of LA was measured at the apical two-chamber plane (Fig. 2C).

**Fig. 3:**
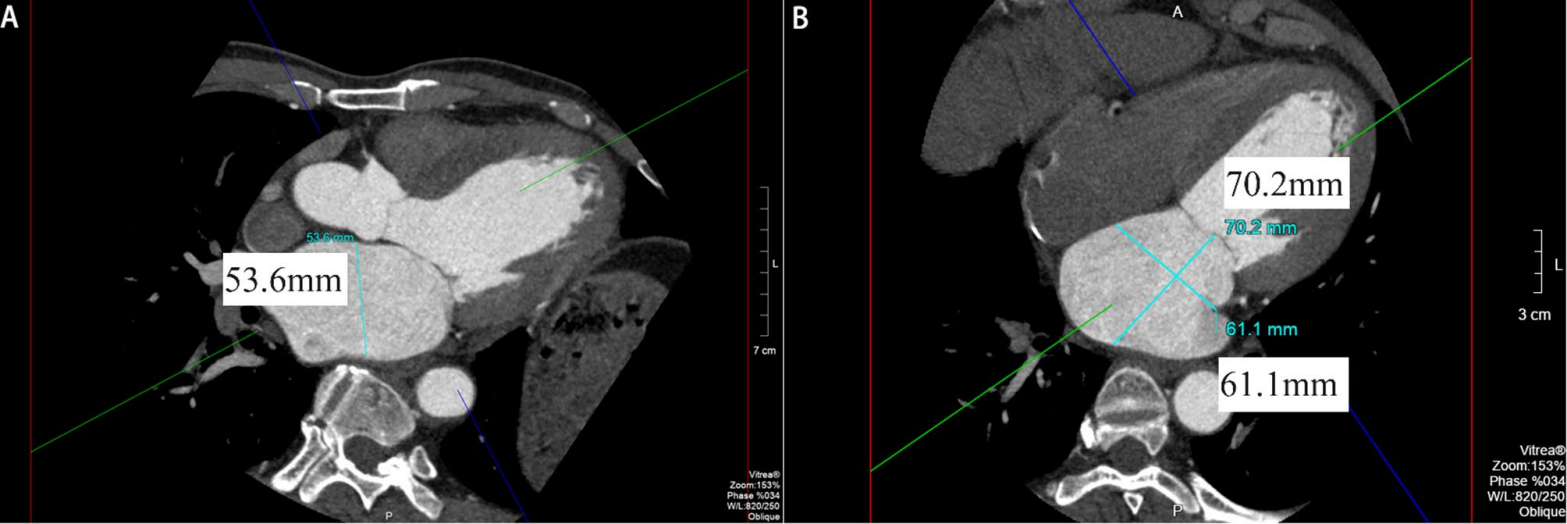
**A**: Echocardiography measurement of the LA anteroposterior diameter at the parasternal left ventricular long axis plane. **B**: Echocardiography measurement of the long diameter and transverse diameter of LA at the four-chamber plane.

Multiplanar reformation (MPR)① image processing method: The parasternal left ventricular long axis plane on echocardiography was manually modified to assess the LA anteroposterior diameter (Fig. 3A), the distance from a vertical line taken from the posterior wall of the aorta to the posterior wall of the left atrium was measured (avoiding the enlarged uncoronal sinus wall and pulmonary vein opening); the LA long diameter (the distance from the midpoint of the mitral ring plane to the top of the left atrium) and the transverse diameter (the distance from the midpoint of the atrial septum to the lateral wall of the left atrium) were measured at the apical four-chamber plane based on the Guidelines of Echocardiography Measurement of Chinese Adults (Fig. 3B).

**Fig. 4:**
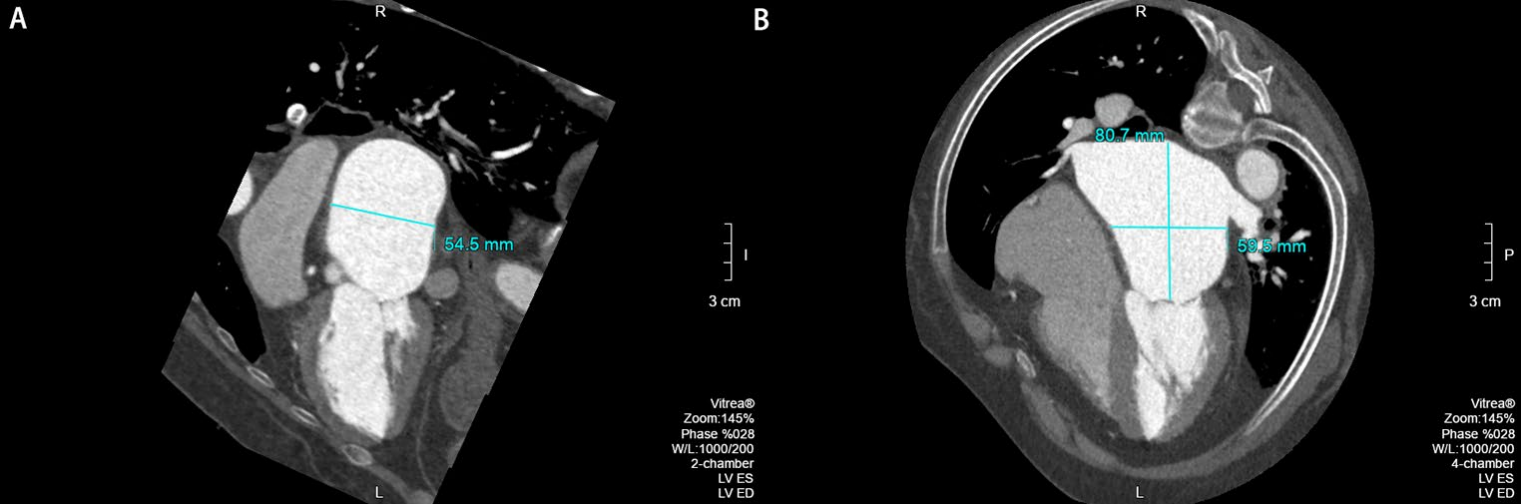
**A**: The anteroposterior diameter of the LA measured at the apical two-chamber plane, on the MPR image. **B**: Measurement of the long diameter and transverse diameter of LA at the apical four-chamber plane on the MPR image

MPR② image processing method: As selected by the system, the LA anteroposterior diameter (the maximum distance between anterior and posterior walls of left atrium) was measured in the apical two-chamber plane (Fig. 4A), while the LA long diameter (the distance from the midpoint of the mitral ring plane to the top of the left atrium)and transverse diameter (the distance from the midpoint of the atrial septum to the lateral wall of the left atrium) were measured at the apical four-chamber plane (Fig. 4B).

**Fig. 5:**
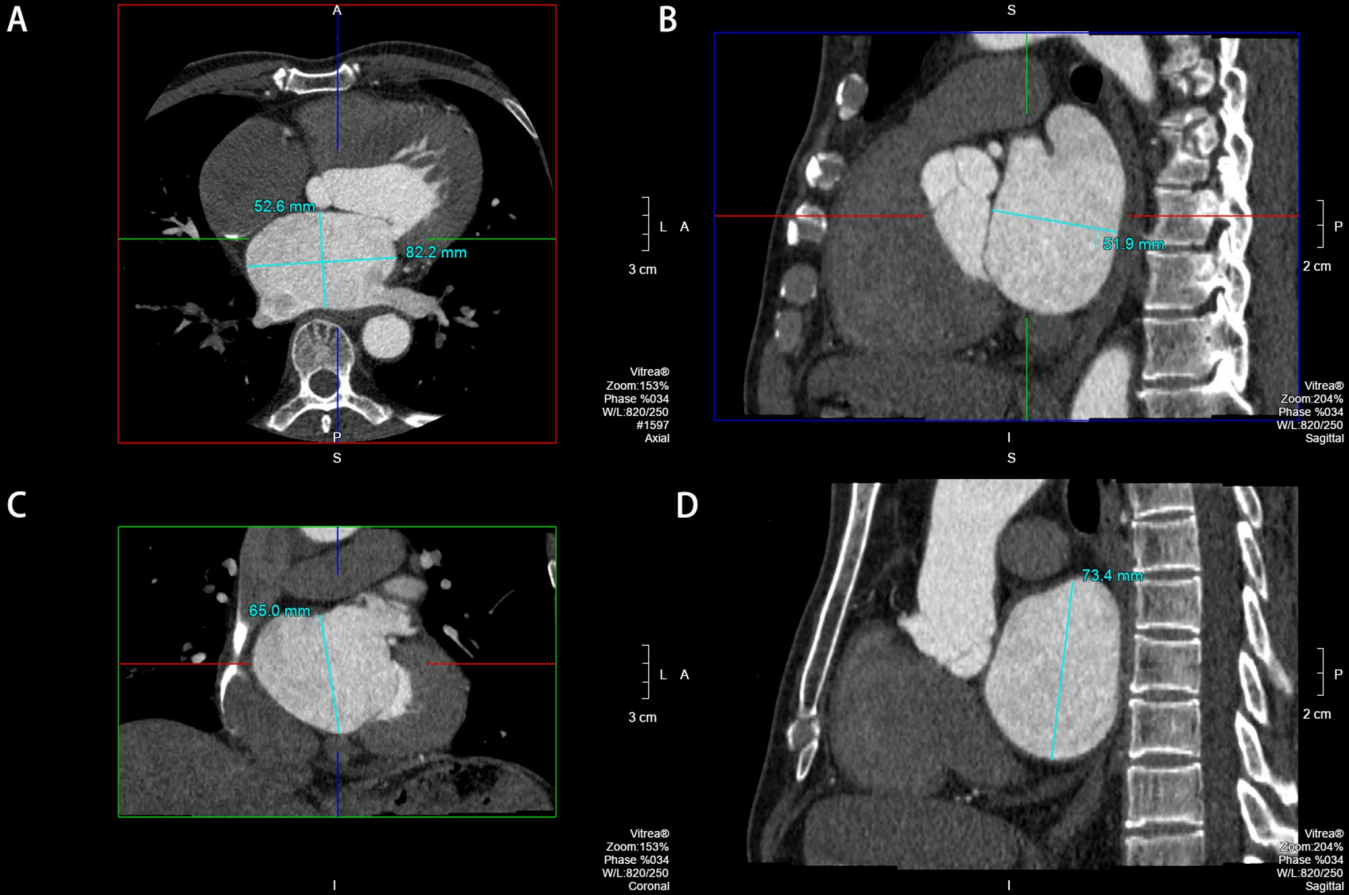
**A**: Measurement of the anteroposterior diameter (represented by MRP③a) and transverse diameter of LA in the maximum axial plane. **B**: Measurement of the LA anteroposterior diameter (represented by MRP③b) at its maximum sagittal plane. **C**: Measurement of the LA long diameter (represented by MRP③a) at the maximum coronal plane. **D**: Measurement of the LA long diameter (represented by MRP③b) at its maximum sagittal plane

MPR③ image processing method: LA diameters were measured on an orthogonal image (Fig. 5) [12]. Measurement of the anteroposterior diameter (represented by MRP③a) and transverse diameter of LA in the maximum axial plane (Fig. 5A), measurement of the LA anteroposterior diameter (represented by MRP③b) at its maximum sagittal plane (Fig. 5B), measurement of the LA long diameter (represented by MRP③a) at the maximum coronal plane (Fig. 5C) and measurement of the LA long diameter (represented by MRP③b) at its maximum sagittal plane (Fig. 5D).

**Fig. 6:**
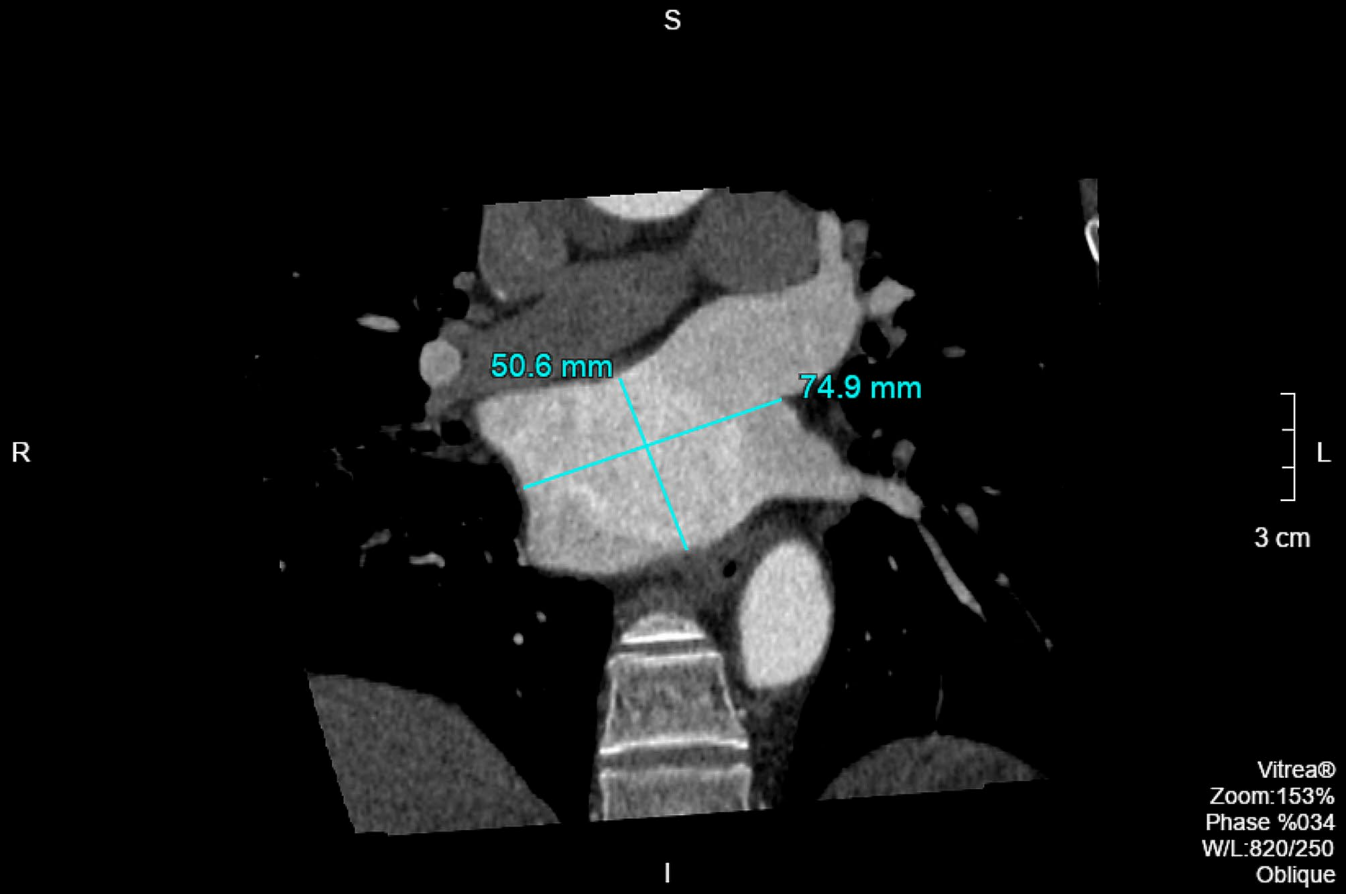
Measurement of the transverse diameter and anteroposterior diameter of LA at an oblique axis plane

MPR④ image processing method: The transverse diameter and anteroposterior diameter of LA were measured at the midpoint of the transverse diameter at the oblique axis plane, which was the distance between the midpoints of the upper and lower left PV and those of the upper and lower right PV on the oblique axis picture (Fig. 6); the horizontal measurement of the LA long diameter, essentially the distance between the top of LA to the mitral orifice, was the same as the MPR① long diameter measurement, therefore it was not remeasured [14]. 

### Image processing time recording method

The time consumed by each measurement, namely t_VR1_, t_VR2_, t_MPR1_, t_MPR2_, t_MPR3_, and t_MPR4_, was recorded; t_VR1_ only included the diameter measurement time in the LA VR processing method ①, and did not include the LA remodeling time, as the LA remodeling was routinely conducted in PV CTA post-processing. The LA long diameter processing time for MPR④ processing method was identical to the LA long diameter processing time for MPR① processing method.

### Image processing

Image data were measured by two imaging diagnostic physicians who have been working for 15 (physician 1) and 2 (physician 2) years and did not know the purpose of the study; one month later, the image data were measured a second time by physician.

### Statistical processing

IBM SPSS Statistics 22.0 (IBM, New York, United States) and Origin2023bSRI software packages were used for statistical analysis of data (OriginLab, Hampton, the US). All measurement data were examined for normal distribution, with those expressing normal distribution represented as mean ± standard deviation, and those non-normally distributed measurement data were expressed as P50 (P25, P75). *P* < 0.05 indicated statistical differences.

The intraclass correlation coefficient (ICC) is the ratio of individual variance to total variance and is often used to evaluate the consistency of the same quantitative measurement results between different measurement methods or different testers. In this study, we used ICC to evaluate the consistency of a measurement of LA diameter between two physicians, between two measurements by the same physician, and between three measurements by two physicians. ICC < 0.2 indicated poor consistency; 0.2 < ICC < 0.4 indicated common consistency; 0.4 < ICC < 0.6 indicated moderate consistency; 0.6 < ICC < 0.8 indicated strong consistency; 0.8 < ICC < 1.0 indicated very strong consistency.

Initially, the homogeneity of variance (Levene method) was performed on the sample means. In the event of variance heterogeneity, the Friedman test for comparison of multiple correlated samples was conducted to determine if the positions of multiple overall distributions were the same, and the *q* test for pairwise comparison of multiple correlated samples was conducted if these positions were different. In the Friedman test, P value represented the level of significance difference, where *P* < 0.05 indicated that there was a significant relationship between the two test results, which meant that the model has a good fitting effect.

## Results

### Baseline data

The image data of 87 patients were included in the study; All the patients were diagnosed with AF, and included 50 males and 37 females, aged 45 ~ 76 years, with a mean age of (60.74 ± 8.70) years; the LA axis was generally consistent with the left ventricular axis in 46 cases, and was inconsistent in 41 cases; LA was in an erect position or semierect position in 27 cases.

### Consistency evaluation of LA diameter measurement by two physicians

The ICC of two measurements of physician 1 (intraclass), the second measurements of physician 1 and physician 2 (interclass), and three measurements of physician 1 and physician 2 were calculated, and the consistency was high or extremely high. Specific results are shown in Table 1.

**Table 1:** Consistency evaluation of the left atrium (LA) diameters measured by the two physicians (95%CI)

Parameters	Measurement 1 by physician 1	Measurement 2 by physician 1	Measurement by physician 2	Consistency of two measurements by physician 1			physician 1-physician 2 Consistency			Consistency of 3 measurements			
				*ICC value*	*F value*	*P value*	*ICC value*	*F value*	*P value*	*ICC value*	*F value*	*P value*	
**Anteroposterior diameter**													
VR①	46.34 ± 10.66	45.51 ± 10.17	45.86 ± 10.24	0.94(0.90 ~ 0.96)	29.72	< 0.001	0.95(0.92 ~ 0.97)	38.52	< 0.001	0.95(0.93 ~ 0.97)	59.77	< 0.001	
VR②	44.30 ± 8.62	45.03 ± 9.34	44.47 ± 8.32	0.83(0.75 ~ 0.88)	10.57	< 0.001	0.84(0.76 ~ 0.89)	11.32	< 0.001	0.86(0.81 ~ 0.90)	19.33	< 0.001	
MPR①	44.39 ± 9.00	43.59 ± 9.21	44.12 ± 8.25	0.90(0.85 ~ 0.94)	19.49	< 0.001	0.90(0.86 ~ 0.94)	19.86	< 0.001	0.92(0.89 ~ 0.94)	35.13	< 0.001	
MPR②	53.24 ± 9.26	51.45 ± 9.08	54.19 ± 9.58	0.75(0.64 ~ 0.83)	7.03	< 0.001	0.81(0.72 ~ 0.87)	9.26	< 0.001	0.82(0.76 ~ 0.87)	14.71	< 0.001	
MPR③a	44.56 ± 9.20	44.85 ± 9.41	45.00 ± 9.78	0.98(0.96 ~ 0.98)	79.12	< 0.001	0.97(0.96 ~ 0.98)	62.72	< 0.001	0.97(0.96 ~ 0.98)	115.29	< 0.001	
MPR③b	44.50 ± 9.26	44.86 ± 9.51	45.00 ± 9.42	0.96(0.94 ~ 0.98)	52.64	< 0.001	0.97(0.95 ~ 0.98)	60.52	< 0.001	0.97(0.96 ~ 0.98)	95.83	< 0.001	
MPR④	35.75 ± 7.91	35.88 ± 6.93	35.19 ± 7.08	0.78(0.68 ~ 0.85)	8.06	< 0.001	0.80(0.71 ~ 0.87)	9.08	< 0.001	0.81(0.75 ~ 0.87)	13.98	< 0.001	
**Long diameter**													
VR①	66.38 ± 7.50	67.89 ± 7.40	66.93 ± 7.48	0.93(0.89 ~ 0.95)	27.04	< 0.001	0.92(0.88 ~ 0.95)	24.50	< 0.001	0.93(0.91 ~ 0.95)	42.91	< 0.001	
VR②	62.99 ± 9.56	63.05 ± 8.65	64.09 ± 8.80	0.77(0.66 ~ 0.84)	7.51	< 0.001	0.78(0.68 ~ 0.85)	7.98	< 0.001	0.81(0.74 ~ 0.86)	13.52	< 0.001	
MPR①	64.33 ± 10.64	66.09 ± 10.80	64.43 ± 10.45	0.83(0.76 ~ 0.89)	11.01	< 0.001	0.76(0.65 ~ 0.83)	7.17	< 0.001	0.82(0.75 ~ 0.87)	14.28	< 0.001	
MPR②	64.78 ± 11.47	64.84 ± 10.49	64.90 ± 11.25	0.91(0.87 ~ 0.94)	21.67	< 0.001	0.92(0.89 ~ 0.95)	25.13	< 0.001	0.93(0.91 ~ 0.95)	43.28	< 0.001	
MPR③a	63.30 ± 8.21	64.71 ± 8.03	64.86 ± 7.87	0.87(0.81 ~ 0.91)	14.30	< 0.001	0.88(0.82 ~ 0.92)	15.83	< 0.001	0.94(0.91 ~ 0.96)	47.97	< 0.001	
MPR③b	67.77 ± 9.82	66.97 ± 8.99	69.80 ± 9.32	0.89(0.84 ~ 0.93)	17.82	< 0.001	0.87(0.80 ~ 0.91)	13.83	< 0.001	0.89(0.85 ~ 0.93)	26.27	< 0.001	
**Transverse diameter**													
VR①a	54.70(48.90,58.00)	54.70(50.10,60.00)	54.00(48.70,58.30)	0.93(0.90 ~ 0.96)	28.37	< 0.001	0.94(0.92 ~ 0.96)	34.96	< 0.001	0.94(0.92 ~ 0.96)	49.04	< 0.001	
VR①b	57.00(50.50,62.10)	55.50(49.10,62.30)	54.00(48.80,60.00)	0.94(0.92 ~ 0.96)	34.98	< 0.001	0.86(0.79 ~ 0.90)	12.77	< 0.001	0.90(0.86 ~ 0.93)	27.04	< 0.001	
VR②	50.20(44.40,56.20)	49.30(45.20,54.50)	50.90(46.60,57.20)	0.84(0.77 ~ 0.90)	11.76	< 0.001	0.82(0.74 ~ 0.88)	9.94	< 0.001	0.86(0.81 ~ 0.90)	19.22	< 0.001	
MPR①	52.88 ± 9.34	52.75 ± 8.33	52.52 ± 8.09	0.87(0.81 ~ 0.92)	14.43	< 0.001	0.81(0.72 ~ 0.84)	9.33	< 0.001	0.84(0.78 ~ 0.88)	16.38	< 0.001	
MPR②	50.00 ± 9.59	50.50 ± 9.18	50.94 ± 8.82	0.94(0.90 ~ 0.96)	29.93	< 0.001	0.93(0.90 ~ 0.95)	27.663	< 0.001	0.94(0.92 ~ 0.96)	48.77	< 0.001	
MPR③	78.00(72.10,83.80)	77.30(70.80,81.90)	79.20(73.70,85.00)	0.92(0.88 ~ 0.95)	24.84	< 0.001	0.94(0.92 ~ 0.96)	34.51	< 0.001	0.94(0.91 ~ 0.96)	45.18	< 0.001	
MPR④	57.10(51.70,64.60)	58.50(51.10,64.90)	58.10(52.00,64.80)	0.94(0.91 ~ 0.96)	33.38	< 0.001	0.94(0.91 ~ 0.96)	34.172	< 0.001	0.95(0.93 ~ 0.96)	55.34	< 0.001	

(*Note* VR① refers to the measurement on the VR image of an independent LA remodeling; VR② refers to the measurements on the VR image of the cardiac valves pattern; MPR① refers to the measurement at the ultrasonic plane; MPR② refers to the measurement at the apical two-chamber and four-chamber plane; MPR③ refers to the measurement at the orthogonal plane; MPR④ refers to the measurement at the oblique axis plane; MPR③a of the anteroposterior diameter means measurement at the orthogonal axis plane; MPR③b of the anteroposterior diameter refers to the measurement at the orthogonal sagittal plane; MPR③a of the long diameter refers to the measurement at the coronal plane; MPR③b of the long diameter refers to the measurement at the sagittal plane; VR①a of the transverse diameter refers to the distance between the inner and outer lateral walls of LA; VR①b of the transverse diameter suggests that the minimum distance of the extension line between the left and right PV orifices represents the transverse diameter.)

### Homogeneity of variance analysis of LA diameter measurement by different methods

The Levene method was used to analyze the homogeneity of variance for LA diameters measured by different methods. The results indicated that the variance was heterogeneous for the anteroposterior diameter, long diameter, and transverse diameter of LA measured by different methods, with *F* and *P* values of the three diameters being 2.16 and 0.045, 3.18 and 0.008, and 2.91 and 0.008, respectively.

### Difference in LA diameter measurement by different methods

**Fig. 7:**
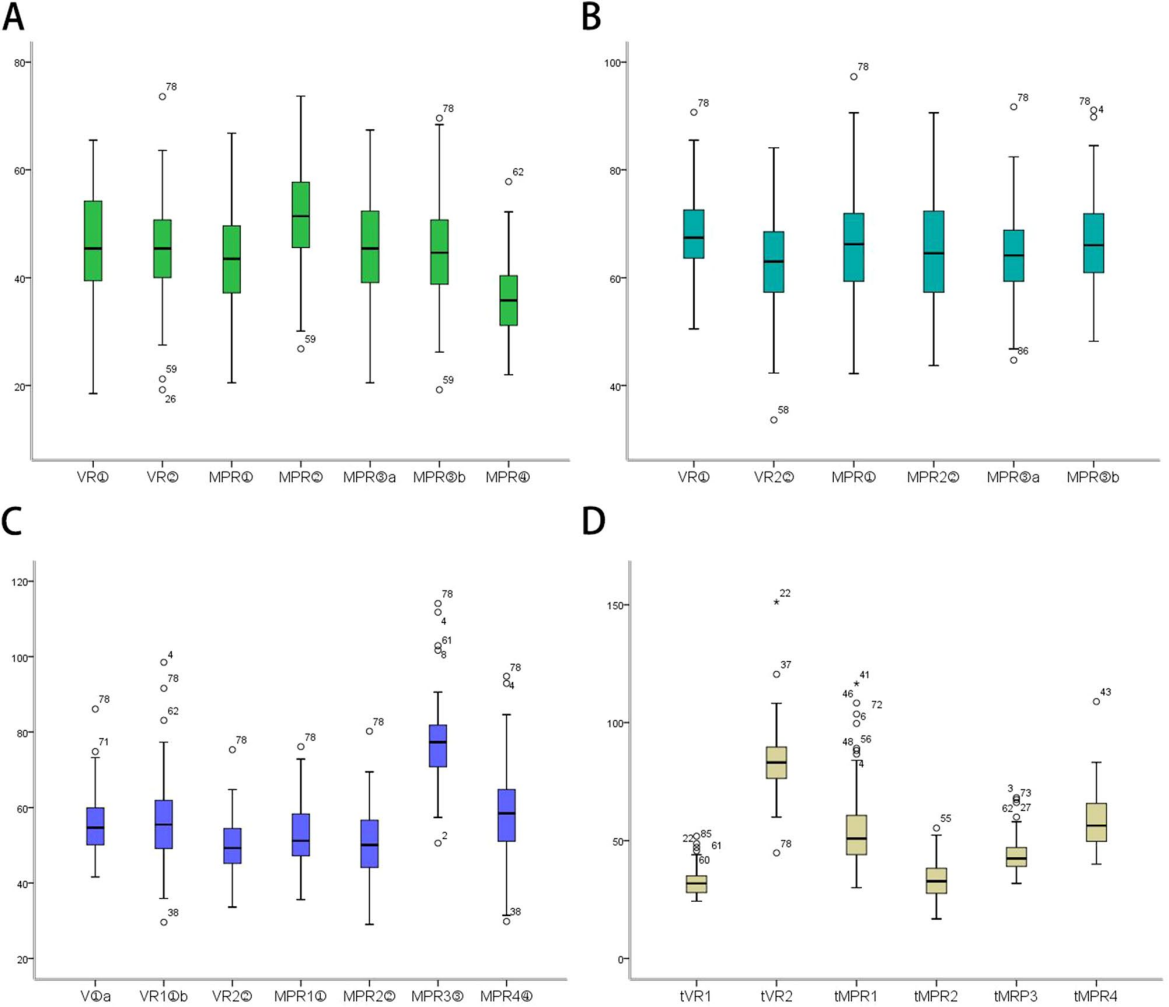
Comparison of different methods for the measurement of LA diameters and consumed time. **A**: anteroposterior diameter; **B**: long diameter; **C**: transverse diameter; **D**: consumed time

Based on the Friedman test, there were statistical differences in the anteroposterior diameter, long diameter, and transverse diameter of LA measured by different methods at *α* = 0.05 (anteroposterior diameter: *χ*^*2*^ = 222.28, *P* < 0.001, long diameter: *χ*^*2*^ = 32.74, *P* < 0.001, transverse diameter: *χ*^*2*^ = 293.83, *P* < 0.001); On the basis of the *q* test for pairwise comparison of multiple correlated samples (Friedman test) and the Friedman test for multiple gradually decreased correlated samples, there were statistically significant differences between the anteroposterior diameter, long diameter, and transverse diameter of LA measured by various methods (Table 2; Fig. 7).

**Table 2:** Friedman test results of pairwise comparison of different methods for measuring the left atrium (LA) diameters on the computed tomography (CT) image

Dimension	Measurement methods		Measurement methods		*q value*	*P value*
	Name	mean ± SD*/* P50 (P25, P75)	Name	mean ± SD*/* P50 (P25, P75)		
Anteroposterior diameter	MPR④	35.88 ± 6.93	MPR①	43.59 ± 9.21	5.95	< 0.001
			VR②	45.03 ± 9.34	7.53	< 0.001
			MPR③b	44.86 ± 9.51	7.67	< 0.001
			MPR③a	44.85 ± 9.41	8.05	< 0.001
			VR①	45.51 ± 10.17	8.21	< 0.001
			MPR②	51.45 ± 9.08	14.14	< 0.001
	MPR①	43.59 ± 9.21	VR②	45.03 ± 9.34	1.58	1.000
			MPR③b	44.86 ± 9.51	-1.72	1.000
			MPR③a	44.85 ± 9.41	-2.11	0.740
			VR①	45.51 ± 10.17	2.26	0.496
			MPR②	51.45 ± 9.08	-8.72	< 0.001
	VR②	45.03 ± 9.34	MPR③b	44.86 ± 9.51	-0.14	1.000
			MPR③a	44.85 ± 9.41	-0.53	1.000
			VR①	45.51 ± 10.17	0.68	1.000
			MPR②	51.45 ± 9.08	-7.14	< 0.001
	MPR③b	44.86 ± 9.51	MPR③a	44.85 ± 9.41	0.39	1.000
			VR①	45.51 ± 10.17	0.54	1.000
			MPR②	51.45 ± 9.08	7.00	< 0.001
	MPR③a	44.85 ± 9.41	VR①	45.51 ± 10.17	0.16	1.000
			MPR②	51.45 ± 9.08	6.62	< 0.001
	VR①	45.51 ± 10.17	MPR②	51.45 ± 9.08	-6.46	< 0.001
Long diameter	VR②	63.05 ± 8.65	MPR③a	64.71 ± 8.03	-0.041	1.000
			MPR②	64.84 ± 10.49	-2.48	0.202
			MPR③b	66.97 ± 8.99	-3.06	0.033
			MPR①	66.09 ± 10.80	-3.57	0.005
			VR①	67.89 ± 7.40	4.24	< 0.001
	MPR③a	64.71 ± 8.03	MPR②	64.84 ± 10.49	2.43	0.226
			MPR③b	66.97 ± 8.99	-3.02	0.038
			MPR①	66.09 ± 10.80	3.53	0.006
			VR①	67.89 ± 7.40	4.19	< 0.001
	MPR②	64.84 ± 10.49	MPR③b	66.97 ± 8.99	-0.59	1.000
			MPR①	66.09 ± 10.80	1.09	1.000
			VR①	67.89 ± 7.40	1.76	1.000
	MPR③b	66.97 ± 8.99	MPR①	66.09 ± 10.80	0.51	1.000
			VR①	67.89 ± 7.40	1.12	1.000
	MPR①	66.09 ± 10.80	VR①	67.89 ± 7.40	0.67	1.000
Transverse diameter	VR②	49.30 (45.20, 54.50)	MPR②	50.10 (44.10, 56.70)	-1.67	1.000
			MPR①	49.30 (46.60, 58.50)	-3.90	0.002*
			VR①b	55.50 (49.10, 62.30)	5.91	< 0.001*
			VR①a	54.70 (50.10, 60.00)	6.56	< 0.001*
			MPR④	58.50 (51.10, 64.90)	-8.49	< 0.001*
Transverse diameter			MPR③	77.30 (70.80, 81.90)	-14.99	< 0.001*
	MPR②	50.10 (44.10, 56.70)	MPR①	49.30 (46.60, 58.50)	0.026	0.543
			VR①b	55.50 (49.10, 62.30)	4.25	< 0.001*
			VR①a	54.70 (50.10, 60.00)	4.90	< 0.001*
			MPR④	58.50 (51.10, 64.90)	-6.83	< 0.001*
			MPR③	77.30 (70.80, 81.90)	-13.32	< 0.001*
	MPR①	49.30 (46.60, 58.50)	VR①b	55.50 (49.10, 62.30)	2.02	0.916
			VR①a	54.70 (50.10, 60.00)	2.67	0.161
			MPR④	58.50 (51.10, 64.90)	-4.60	< 0.001*
			MPR③	77.30 (70.80, 81.90)	-11.09	< 0.001*
	VR①b	55.50 (49.10, 62.30)	VR①a	54.70 (50.10, 60.00)	0.65	1.000
			MPR④	58.50 (51.10, 64.90)	-2.58	0.208
			MPR③	77.30 (70.80, 81.90)	-9.07	< 0.001*
	VR①a	54.70 (50.10, 60.00)	MPR④	58.50 (51.10, 64.90)	-1.93	1.000
			MPR③	77.30 (70.80, 81.90)	-8.42	< 0.001*
	MPR④	58.50 (51.10, 64.90)	MPR③	77.30 (70.80, 81.90)	6.49	< 0.001*

(*Note* VR① refers to the measurement on the VR image of an independent LA remodeling; VR② refers to the measurements on the VR image of the cardiac valves pattern; MPR① refers to the measurement at the ultrasonic plane; MPR② refers to the measurement at the apical two-chamber and four-chamber plane; MPR③ refers to the measurement at the orthogonal plane; MPR④ refers to the measurement at the oblique axis plane; MPR③a of the anteroposterior diameter means measurement at the orthogonal axis plane; MPR③b of the anteroposterior diameter refers to the measurement at the orthogonal sagittal plane; MPR③a of the long diameter refers to the measurement at the coronal plane; MPR③b of the long diameter refers to the measurement at the sagittal plane; VR①a of the transverse diameter refers to the distance between the inner and outer lateral walls of LA; VR①b of the transverse diameter suggests that the minimum distance of the extension line between the left and right PV orifices represents the transverse diameter.)

### Difference in time consumed by different methods for LA diameter measurement

Based on the rank sum test of randomized block design (Friedman test), there were statistical differences in the time required by different LA diameter measurement methods at *α* = 0.05 (*χ*^*2*^ = 333.10, *P* < 0.001); based on the Friedman test for multiple gradually decreased correlated samples, there were statistical differences in the time required by different LA diameter measurement methods (Table 3; Fig. 7).

**Table 3:** Mean rank and homogeneous subset results of Friedman test of different methods for measuring gradually decreased the left atrium (LA) diameters

Anteroposterior diameter	Subsets				Long diameter	Subsets		Transverse diameter	Subsets					
	1	2		3		1	2							
MPR④	1.56				VR②	2.87		VR②	2.06					
MPR①		3.51			MPR③a	2.88		MPR②		2.60				
VR②		4.03			MPR②	3.57	3.57	MPR①			3.33			
MPR③a		4.07			MPR③b		3.74	VR①b				3.99		
MPR③b		4.20			MPR①		3.88	VR①a				4.21		
VR①		4.25			VR①		4.07	MPR④					4.84	
MPR②			6.37					MPR③						6.97
*q value*		9.48			*q value*	5.59	2.70	*q value*				0.74		
*P value*		0.070			*P value*	0.118	0.581	*P value*				0.824		

(*Note* VR① refers to the measurement on the VR image of an independent LA remodeling; VR② refers to the measurements on the VR image of the cardiac valves pattern; MPR① refers to the measurement at the ultrasonic plane; MPR② refers to the measurement at the apical two-chamber and four-chamber plane; MPR③ refers to the measurement at the orthogonal plane; MPR④ refers to the measurement at the oblique axis plane; MPR③a of the anteroposterior diameter means measurement at the orthogonal axis plane; MPR③b of the anteroposterior diameter refers to the measurement at the orthogonal sagittal plane; MPR③a of the long diameter refers to the measurement at the coronal plane; MPR③b of the long diameter refers to the measurement at the sagittal plane; VR①a of the transverse diameter refers to the distance between the inner and outer lateral walls of LA; VR①b of the transverse diameter suggests that the minimum distance of the extension line between the left and right PV orifices represents the transverse diameter.)

## Discussion

### Main discovery of LA diameter measurement

In this study, (1) the consistency of LA diameter measurements between the two physicians was either strong or very strong. (2) The LA diameter estimated using different methods was not identical. (3) LA diameter measurement on VR① and MPR② images required less time compared to LA diameter measurement on other images.

The LA anteroposterior diameter is the maximum vertical diameter of anterior and posterior walls of LA. The LA long diameter is the distance between the midpoint of the mitral annulus plane and the top of LA. The LA transverse diameter is the distance between the midpoint of the atrial septum and the side wall of LA, and it is perpendicular to the long diameter of the LA. Theoretically, all of the diameters measured by VR①, VR②, MPR①, and MPR② methods meet the anatomical definition.

### Discussion of measurement methods for LA Anteroposterior diameter

MPR③a and MPR③b were used to measure the maximum distance between the anterior and posterior walls at the maximum axial plane of LA in the orthogonal plane or sagittal plane, and MPR④ were used to measure the distance between the upper left and right PV midpoint and the lower left and right PV midpoint [14]. The LA had an uneven funnel form. Theoretically, the diameter measured by the two methods is not the anatomically localized LA anteroposterior diameter. There were no statistically significant differences in the diameter measured by MPR③ and by VR①, VR②, and MPR①, which only indicated that the measured result was comparable to the actual anteroposterior diameter of the LA. MPR④ yielded the smallest anteroposterior diameter for the LA, indicating that this approach did not accurately assess the anteroposterior diameter.

The LA anteroposterior diameter measured by MPR② was the largest and had the lowest ICC, which may be associated with the inconsistency of LA with the left ventricular axis [16]. In addition, the LA axis was inconsistent with the left ventricular axis in 41 cases (41/87) in this group, whereas the default apical two-chamber plane was the anteroposterior diameter plane of the left ventricle, which may be the oblique anteroposterior diameter plane of LA. Therefore, the LA anteroposterior diameter measured at this plane was larger. Meanwhile, different physicians have different cognitive levels, and some of them may shift the measurement plane to the LA anteroposterior diameter plane depending on their own experience. However, some physicians also measure diameters at the default plane, hence, this measurement method has the lowest ICC in the consistency evaluation of measurement for these physicians and is not suitable for promotion.

VR② for the LA anteroposterior diameter was similar to the MPR② method, in which the LA anteroposterior diameter was assessed on the MPR image. In the VR② method, the anatomical structure was more easily observed, and thus the LA anteroposterior diameter measured by this method was statistically different from those measured by MPR①, MPR③b, MPR③a, and VR① (*P* > 0.05); the ICC of this method was slightly higher than that of MPR②, but lower than that of other methods. However, this method is time-consuming and therefore not suitable for promotion.

MPR① was compatible with the LA anteroposterior diameter measurement method reported by Stolzmann et al. and Stojanovska et al., that is, the LA anteroposterior diameter was measured at the parasternal left ventricular long axis plane on echocardiography [17–19]. The LA anteroposterior diameter determined by this method and by VR②4, MPR③, MPR③a, and VR①4 did not differ statistically (*P* > 0.05), and the ICC was greater than 0.9. However, this method is more complex and time-consuming than VR①, MPR②, and MPR③, and requires physicians to make adjustments in several aspects (*P* < 0.001), hence it is not ideal for clinical promotion.

On the VR image of the separate LA remodeling, VR① was used to measure the maximum anteroposterior diameter of LA, which was similar to the LA measurement method provided by Seewöster et al. [20] The difference was that Seewöster et al. measured the diameter based on the MPR image, however our method is more intuitive. In addition, (1) the LA anteroposterior diameter determined by this method is similar to that measured by VR②4, MPR③b, MPR③a, and MPR① (*P* > 0.05). (2) The ICC of this method is the second highest, behind that of MPR③. (3) This method required the least time, and thus has a high promotion value.

### Discussion of measurement methods for LA long diameter

MPR③a and MPR③b were used to measure the maximum coronal plane of LA at the orthogonal plane and the maximum height of LA at the sagittal plane. Theoretically, the diameter measured by this method, is not the anatomically localized LA anteroposterior diameter. In addition, the LA long diameter assessed by MPR③a was smaller than that by MPR① and VR① (*P* = 0.006, *P <* 0.0001); however, there were no statistically significant differences between the LA long diameter measured by MPR③b and that measured by MPR①, MPR② and VR①. The reason may be that the top of LA and the mitral annulus are distant from one another in the coronal plane, yet coincide in the sagittal plane. In addition, the ICC of the diameter measured with MPR③a and MPR③b was less than that of the diameter recorded with VR① and MPR②. Therefore, they do not qualify for clinical promotion.

The LA long diameter measured by VR② was the shortest with the least mean rank in this study. It exhibited the lowest ICC, which may have been due to the fact that LA axis and the left ventricular axis were not on the same line [16]. In addition, 27 patients were in an upright position or semierect position, therefore the LA long diameter assessed at the apical four-chamber plane may have been the oblique diameter between the anterior and posterior walls of LA rather than the actual LA long diameter, resulting in the lowest mean rank. At the same time, one of the physicians may have adjusted the plane at which the LA was measured, while the other did not, resulting in a measurement result with the lowest ICC. Therefore, this approach has minimal clinical relevance.

MPR② was comparable to VR② and the method used by Shiro et al. [21], with the exception that MPR2 is based on the MPR image and VR2 is based on the VR image. MPR② has similar deficiencies to VR②, and there are no statistical differences in the LA long diameter measured by the two methods. Therefore, they do not qualify for clinical promotion.

MPR① is essentially the same as in LA long diameter measurement, with the exception that MPR① is based on the manually adjusted plane while MPR② is based on the default plane. The ICC was therefore low. Thus, they do not qualify for promotion.

In the VR① method, the diameter was measured at the separately created sagittal plane or coronal plane of LA, which was determined by the line connecting the peak of LA to the midpoint of mitral annulus. This procedure is similar to the method used by Seewöster et al. [20]. There were no statistically significant differences in the LA long diameter measured by this method and by MPR② and MPR①, indicating the reliability of the results measured by this method; it had the highest ICC, indicating the excellent repeatability of this method, and it required the least time. Therefore, it has high clinical application value.

### Discussion of measurement methods for LA transverse diameter

In the MPR③ measurement method, the greatest distance between the inner and outer lateral walls of LA was measured at the maximum axial plane [12]. This method measures the transverse oblique diameter rather than the transverse diameter of LA, which is theoretically bigger than the LA transverse diameter. Moreover, in this study, the LA transverse diameter measured by this method was also the largest. Therefore, this method is not suitable for clinical promotion.

MPR④ and VR①b, two similar measurement methods, were used to measure the distance between two PV midpoints, with the difference being that the former was based on the MPR image and the latter was based on the VR image, which was consistent with the method used by Shimamoto et al. [14] Anatomically, the LA transverse diameter estimated using this method is not accurate. Another four PV lines are varied obviously, which can impact the precision of the measurement data. This method is therefore not suitable for clinical promotion.

Similar to the method used by Shiro et al., MPR①, MPR②, and VR②, are essentially the same measurement methods, that is, the LA transverse diameter is measured at the cardiac chamber plane [21]. The difference is that MPR① and MPR② are derived from the MPR image and VR② is based on the VR image. Anatomically, the LA is irregularly funnel-shaped, and its diameter deviates to varying degrees. In the three methods, the diameter is measured from the center of the atrial septum to the outer lateral wall (left lateral wall) of LA. However, due to the large variance in the outer lateral wall, such as the slope shape of the outer lateral wall when the LA anteroposterior diameter is small, the distance from the atrial septum to the posterior wall of LA is often measured as the transverse diameter and the result is short. In this study, when comparing the LA transverse diameter determined by the three methods using the Friedman test for samples that consistently decreased in size, it was found to be smaller than the measurement taken from the VR image. The three methods also had a strong ICC (ICC > 0.8), which indirectly reflected the differences in the mean rank of the LA transverse diameter measured by the three methods. Therefore, it is difficult to clinically use the three methods to assess the LA transverse diameter.

The VR①a measurement method, in which the LA transverse diameter is assessed on the separate VR image of LA, is similar to the method proposed by Seewöster et al. [20]. The results of this study revealed that there were no statistically significant differences in the LA transverse diameter measured by VR①a and by MPR①, but the mean rank of the former was larger than that of the latter in the Friedman test for gradually decreasing samples. This measurement used clear anatomical landmarks, and the measured LA transverse diameter was slightly larger, indicating that the result measured by this method should be closer to the actual LA transverse diameter. In addition, the ICC of this method was similar to that of MPR②, but greater than those of VR② and MPR①, indicating that this method is repeatable. This method also required the least time. Therefore, it has a higher clinical promotion value.

There are some limitations in this study. ① The results were not compared with echocardiographic data, as only one anteroposterior diameter was measured on echocardiography in the collected cases, which was of little significance to compare; individual data were also used for ultrasonic LA three-dimensional reconstruction, but the data measured by small-sample three-dimensional cardiac ultrasound were not compared with those by CT due to the ineffectiveness of the reconstruction. ② Artificial intelligence is being employed for multivessel measurement and prediction of disease [22], which may be the direction of our future development and efforts while we adopt measurements.

## Conclusion

The LA diameter measured by different methods is different. Analysis of data in this study revealed that the LA diameter data measured on the directly reconstructed VR image of the LA could be immediately located and measured according to the anatomical landmark, hence, the results were closer to the actual anatomical results. Based on the comprehensive evaluation from four factors, namely conformity with the anatomical measurement, accuracy of measurement, consistency evaluation of measurers, and measurement time, the VR① method, in which the LA diameter is measured separately on the VR image, is the most appropriate for clinical application.

## Data Availability

Data related to the current study are available from the corresponding author on reasonable request.

## Abbreviations

PV: pulmonary vein
LA: left atrium
ICC: intraclass correlation coefficient
AF: atrial fibrillation
CT: computed tomography
MRI: magnetic resonance imaging
PV: pulmonary vein
CTA: computed tomography angiography
VR: volume rendering
