# Correction: Evaluating the consistency in different methods for measuring left atrium diameters

**DOI:** 10.1186/s12880-024-01264-x

**Published:** 2024-04-08

**Authors:** Jun-Yan Yue, Kai Ji, Hai-Peng Liu, Qing-Wu Wu, Chang-Hua Liang, Jian-Bo Gao

**Affiliations:** 1 Department of Radiology, The First Affiliated Hospital of Xinxiang Medical University, Weihui Henan Province, 453200 Xinxiang, China; 2 Department of Radiology, The First Affiliated Hospital of Zhengzhou University, No. 1 Jianshe East Road, Erqi District, 450000 Zhengzhou, Henan Province China; 3 Heart Center, The First Affiliated Hospital of Xinxiang Medical University, 453200 Henan Province, Weihui, China

Correction: Yue *et al. BMC Medical Imaging* (2024) 24:57.

10.1186/s12880-024-01231-6

In the original article [1], Figure 3 was incorrectly duplicated from Figure 6 during the typesetting process.

The wrong Figure 3 is shown below:


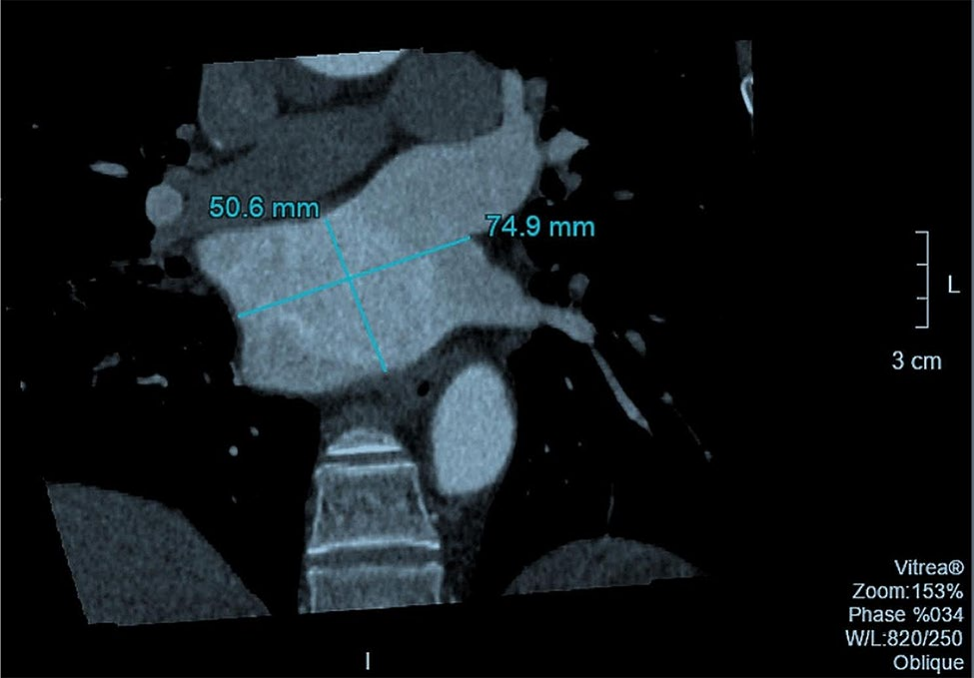


The correct Figure 3 is shown below:


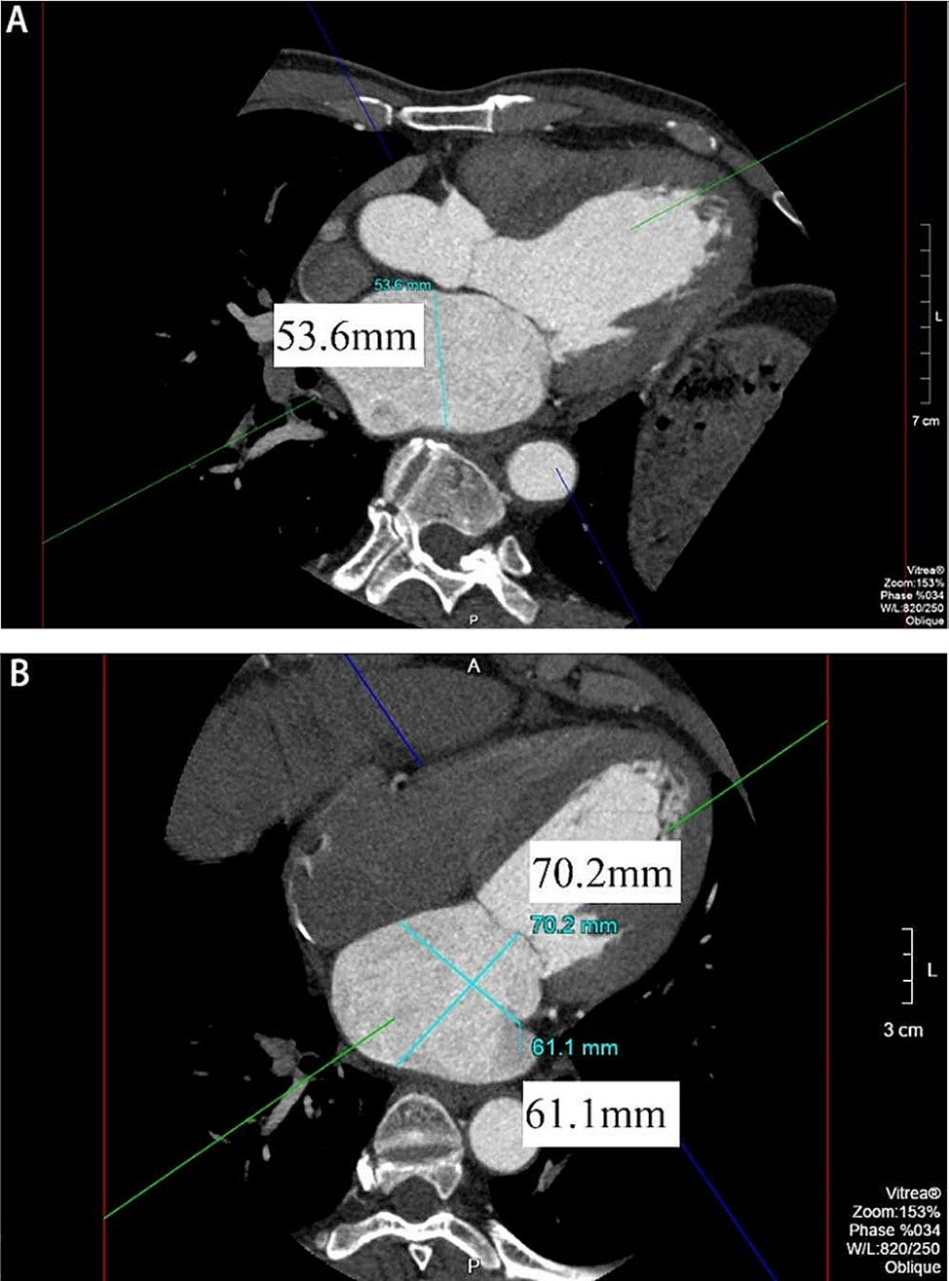


The original article [1] has been corrected.
